# 1α,25-dihydroxyvitamin D3 Attenuates TGF-β-Induced Pro-Fibrotic Effects in Human Lung Epithelial Cells through Inhibition of Epithelial–Mesenchymal Transition

**DOI:** 10.3390/nu9090980

**Published:** 2017-09-06

**Authors:** Fei Jiang, Yong Yang, Lian Xue, Bingyan Li, Zengli Zhang

**Affiliations:** 1 Department of Labor Hygiene and Environmental Health, School of Public Health of Soochow University, 199 Renai Road, Suzhou 215123, China; jiangfei527@suda.edu.cn (F.J.); foreverlove04@126.com (Y.Y.); xyc_celia@163.com (L.X.); 2 Department of Nutrition and Food Hygiene, School of Public Health, Soochow University, 199 Renai Road, Suzhou 215123, China; bingyanli@suda.edu.cn

**Keywords:** vitamin D, fibrosis, EMT

## Abstract

Pulmonary fibrosis is a progressive fibrotic lung disease of persisting lung injury and ineffective wound repair, with poor prognosis. Epithelial–mesenchymal transition (EMT) of alveolar epithelia cells is an early event in the development of pulmonary fibrosis, and transforming growth factor β (TGF-β) is an acknowledged inducer of EMT. Epidemiological studies demonstrated that serum levels of 25-hydroxy-vitamin D were associated with the presence of fibrosis diseases. We investigated whether vitamin D attenuated TGF-β-induced pro-fibrotic effects through inhibiting EMT in human alveolar epithelia A549 cells. A549 cells were cultured with TGF-β alone or in combination with 1α,25-dihydroxyvitamin D3 (1α,25(OH)_2_D_3_). TGF-β increased the expression of the mesenchymal markers (N-cadherin and Vimentin), and decreased the expression of epithelial markers (E-cadherin). 1α,25(OH)_2_D_3_ attenuated these TGF-β-induced alterations. Furthermore, the EMT-related transcription factors (Snail and β-catenin) and the extracellular matrix genes (Collagen I and fibronectin) were inhibited by 1α,25(OH)_2_D_3_, while the expression of vitamin D receptor (VDR) was elevated. In addition, 1α,25(OH)_2_D_3_ alleviated the cell migration and the invasion abilities in TGF-β-stimulated A549 cells, determined by the scratch wound healing and transwell assays. Our findings suggested that 1α,25(OH)_2_D_3_ inhibited the pro-fibrotic phenotype of lung epithelial cells under TGF-β stimulation and provided new clues in the clinical management of pulmonary fibrosis.

## 1. Introduction

Pulmonary fibrosis is a highly heterogeneous and lethal pathological process with limited therapeutic options [1]. It is assumed that pulmonary fibrosis was due to chronic inflammation and disordered wound healing in response to the damage induced by a variety of agents, such as viral infection and radiotherapy or environmental toxins (silicon, quartz) [2]. Pulmonary fibrosis is characterized by the accumulation of myofibroblasts and excessive deposition of the extracellular matrix. Myofibroblasts are derived from a variety of sources including resident mesenchymal cells, bone marrow progenitors (also called as fibrocytes), and epithelial cells undergone epithelial–mesenchymal transition (EMT) [1].

EMT is a complex process in which epithelial cells are converted into mesenchymal cell phenotype with enhancing migration and invasion capacity [3], which has been proved to play an important role in the pathogenesis of fibrotic disease occurring in kidneys, the liver, intestines and lungs [4,5,6,7]. Transforming growth factor β (TGF-β) is a multifunctional cytokine which has profound effects by regulating cell proliferation, differentiation, apoptosis, and extracellular matrix production [8]. Numerous studies have shown that TGF-β plays a critical role in the development of many diseases through the induction of EMT [9,10]. TGF-β, produced by multiple cell types, has been found significantly increased in the idiopathic pulmonary fibrosis patients and animal models [11,12]. Accordingly, the process of EMT induced by TGF-β, has been regarded as an attractive therapeutic target to control fibro-proliferative diseases including pulmonary fibrosis.

Vitamin D is a kind of fat-soluble vitamin, which is responsible for the absorption of calcium, maintenance of phosphate homeostasis and regulation of bone remodeling [13,14,15]. Recent studies have revealed that vitamin D exhibits a wide spectrum of activities in models of inflammations, cancers and diabetes. Epidemiological studies demonstrated that serum levels of 25-hydroxy-vitamin D were associated with fibrosis disease in multiple organs [16,17]. Supplementation with vitamin D or its analogues suppressed renal and liver fibrosis [18,19]. What triggers this anti-fibrotic effect might involve the attenuation of TGF-β. Studies have revealed that vitamin D can reduce the TGF-β expression and attenuate TGF-β-induced EMT in renal tissue, myocardium, as well as the bleomycin-induced lung fibrosis [20,21,22,23,24,25]. Interestingly, vitamin D could potentiate the immunotherapy effect by activating TGF-β [26,27,28,29], suggesting the distinct mechanism might be involved in [30]. Thus, it is essential to investigate the exact role of vitamin D and TGF-β in lung fibrosis.

1α,25(OH)_2_D_3_ is the active form of vitamin D and exerts function via vitamin D receptor (VDR). It is reported that administration of VDR agonist reduced collagen deposition and fibrotic gene expression in mouse models with hepatic injury, while VDR knockout mice developed liver fibrosis spontaneously [31]. A subset of VDR binding sites (VDRE) may be responsible for the control of gene expression. However, many genes regulated by 1α,25(OH)_2_D_3_ do not contain VDRE. Increasing evidence suggests that 1α,25(OH)_2_D_3_ has the capacity to interact with membrane-based signaling, such as Wnt, TGF, EGF, and others [32]. Moreover, TGF-β also exerts profound effects through interaction with multiple signaling. Hence, we investigated the potential mechanism underlying in the anti-fibrotic effect of vitamin D.

Concerning the anti-fibrotic effect of vitamin D in lung fibrosis, Ramirez et al have reported that vitamin D opposed the TGF-β mediated tissue remodeling responses in cultured rat lung fibroblasts [33]. Besides, vitamin D was capable of alleviating the EMT markers induced by TGF-β in rat lung epithelial cells [33]. Ramirez’s work in rat lung fibroblasts and epithelia suggested a pathway capable of influencing TGF-β-related pro-fibrotic responses. Based on Ramirez’s work, we further examined the role of 1α,25(OH)_2_D_3_ in inhibiting cell migration and invasion abilities induced by TGF-β in human alveolar epithelia A549 cells, and investigated the potential mechanisms involved. Importantly, we incubated the cells with 50 nM 1α,25(OH)_2_D_3_—A much lower concentration compared with the previous work of Ramirez. Our findings suggested that the moderate dosage of 50 nM 1α,25(OH)_2_D_3_ could attenuate the pro-fibrotic effects of TGF-β in human alveolar epithelia cells.

## 2. Materials and Methods 

### 2.1. Cell Culture and Treatment

Human type II alveolar epithelia cells A549 cells (lung adenocarcinoma), were obtained from Type Culture Collection of the Chinese Academy of Sciences (Shanghai, China), and maintained in Dulbecco’s modified Eagle’s medium (DMEM, Thermo Fisher Scientific, Waltham, MA, USA) containing 10% fetal bovine serum (FBS), 100 U/mL penicillin, 100 μg/mL streptomycin in a humidified atmosphere of 5% CO_2_ at 37 °C. A549 cells were cultured in 100-mm dishes at a density of 1 × 10^5^ per well. When cells reached 40–50% confluence, the medium was replaced with serum-free DMEM. After starvation for 24 h, cells were treated with 5 ng/mL TGF-β for 72 h. Then, the morphological alteration of treated cells was observed under a microscope.

### 2.2. Western Blot

After being treated, cells were harvested and lysed for total cellular protein using radio-immunoprecipitation assay (RIPA) lysis buffer (Beyotime, Shanghai, China). The cell lysates were centrifuged at 12,000 rpm and 4 °C for 30 min to extract the total cellular protein. The protein concentrations were quantified using bicinchoninic acid (BCA) protein assay kit (Beyotime). Equivalent amount of each protein samples (30 μg) mixed with loading buffer were boiled in water bath and loaded into 10% SDS-PAGE. Separated proteins were transferred onto PVDF membranes. After being blocked with 5% skimmed milk, the membranes were incubated with primary antibodies of E-cadherin, Vimentin, Snail, β-catenin and VDR at 4 °C overnight. Membranes were washed 3 times by PBST and incubated with anti-mouse or anti-rabbit horseradish peroxidase-conjugated antibodies for 1 h. Then, the intensity representing protein expression was visualized by chemiluminescence system.

### 2.3. Immunofluorescence Staining

For immunofluorescence, cells were plated into 24-well plates and allowed to grow until they reached an optimal density. After incubation with TGF-β and/or 1α,25(OH)_2_D_3_ for 72 h, cells were washed with PBS and fixed with 4% paraformaldehyde for 20 min, permeabilized with 0.1% Trition X-100 (Sigma-Aldrich, St. Louis, MO, USA) in PBS for 1.5 h and blocked with 0.5% FBS in PBS for 1 h. The fixed cells were incubated with primary antibodies of E-cadherin and Vimentin overnight at 4 °C. Then, the cells were stained with 647-conjugated secondary antibodies in the dark room for 1.5 h and washed with PBS 3 times. Nuclei were labeled with 4,6-diamidino-2-phenylindole (DAPI) for 20 min. Fluorescent labeling was analyzed using a Confocal Laser Scanning Microscope (TCS SP2, Leica, Wetxlar, Germany).

### 2.4. Scratch Wound Healing Assay

A scratch wound healing assay was performed to test cell migration. A549 cells were seeded into 6-well plates and allowed to form a confluent monolayer. When cells reached 80% confluence, cross lines were made using a 1000 μL sterile pipette tip. The cells were washed three times with sterile PBS to remove the scratched cells. The cells were continuously cultured in 0.5% FBS DMEM medium containing with TGF-β and/or 1α,25(OH)_2_D_3_ for 48 h. Pictures were taken on a phase contrast light microscope at 0, 12, 24 and 48 h using the orientation line to ensure that the identical spots were same. Experiments were conducted 3 times independently.

### 2.5. Invasion Assay

Matrigel (Invitrogen, CA, USA) was transferred from −20 °C to 4 °C for 12 h to obtain a liquid state for use. The upper chamber surface of the basement membrane of the transwell (Corning, NY, USA) was coated with 80 μL mixture of matrigel and medium (dilution ratio is 1:7) and dried at room temperature. After treated as indicated, cells were trypsined, centrifuged, and resuspended in 0.5% FBS DMEM medium at a density of 1 × 10^6^/mL. Then, 100 μL cell suspensions were added to the upper chamber. The bottom chamber was filled with 500 μL of culture medium. After incubation at 37 °C in a humid atmosphere for 24 h, the chamber was removed and cells were washed with PBS, fixed with 4% paraformaldehyde, and stained with methyl violet for 15 min. Cells on the upper surface of the chamber were wiped using a cotton swap. Cells that migrated through the filters were counted under the microscope (×40, CKX41F, Olympus, Tokyo, Japan). Experiments were conducted independently 3 times.

### 2.6. RNA Isolation and Real-Time Reverse Transcription Quantitative PCR

Total RNA of cells was isolated using Trizol reagent (Invitrogen) according to the manufacturer’s instructions. For first-strand complementary DNA (cDNA) synthesis, total RNA was reverse-transcribed using the Transcriptor First Strand cDNA Synthesis Kit (Roche, Basel, Switzerland). Quantitative PCR (qPCR) reactions were conducted with the SYBR Green I Nucleic AcideGel stain (Roche). Reaction parameters were 95 °C for 10 min, followed by 45 cycles of 95 °C for 10 s, 60 °C for 20 s, and 72 °C for 30 s. Fold changes of each gene were calculated by a comparative threshold cycle (Ct) method using the formula 2^−(ΔΔCt)^. GAPDH was used as normalization control. Primer sequences are available (Table 1).

### 2.7. Statistical Analysis

Data were analyzed using SPSS 17.0 (SPSS, Inc., Chicago, IL, USA) and presented as Mean ± Standard Deviation (SD). Student’s *t* test and one-way analysis of variance were used to compare the differences between groups. *p* value < 0.05 was considered statistically significant.

## 3. Results

### 3.1. TGF-β Induces Morphological Alteration in A549 Cells

**Figure 1:**
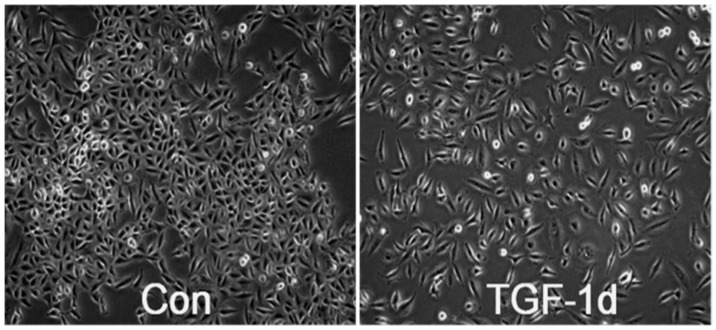

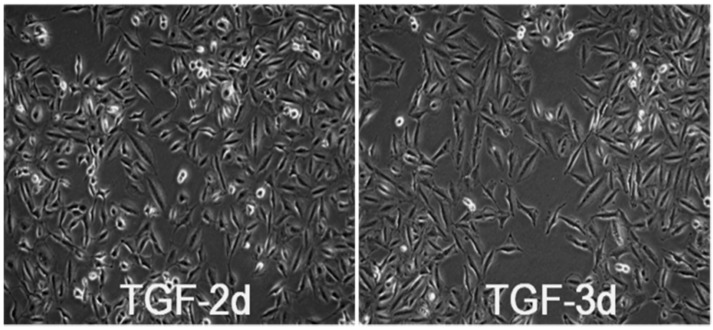
TGF-β induces morphological alteration in A549 cells. A549 cells were exposed to 5 ng/mL TGF-β for 0, 1, 2, 3 days, and morphological features were separately taken at the last day (400× magnification).

In order to establish an effective EMT model and to characterize the effects of TGF-β-induced EMT in A549 cells, morphological features and EMT markers expressions were performed at different time points. Under basal condition, A549 cells displayed a cobbles-toned-like morphology, while 5 ng/mL TGF-β-treated A549 cells were elongated and became more fibroblast-like or spindle-shaped (Figure 1). These results indicated that TGF-β can induce morphological alteration in A549 cells. Similar findings were also observed in human bronchial epithelial BEAS-2B cells (Figure S1A).

### 3.2. TGF-β Induces the Alteration of EMT Markers in A549 Cells

**Figure 2:**
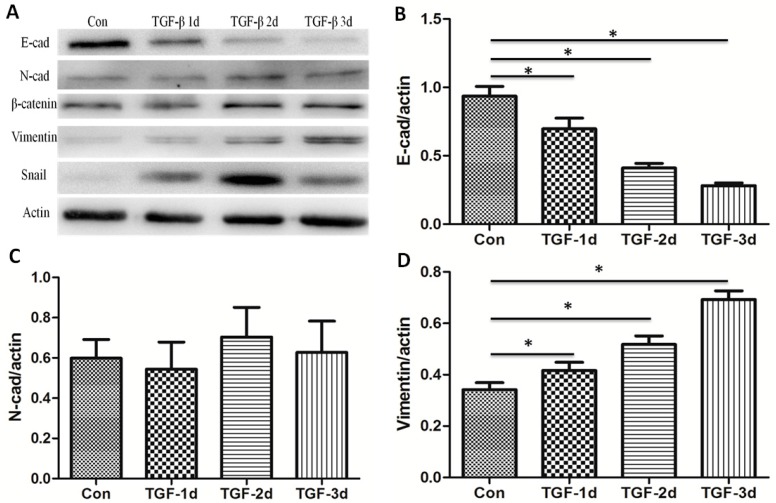

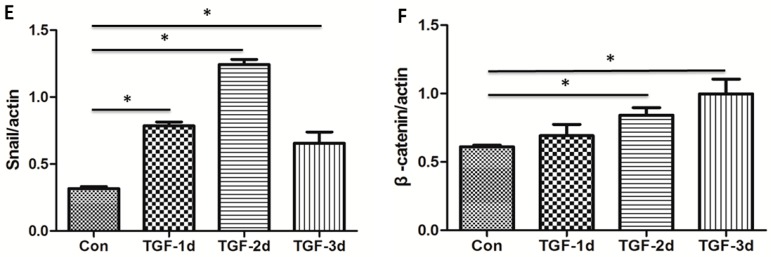
TGF-β induces the alteration of EMT-related genes in A549 cells. A549 cells were exposed to 5 ng/mL TGF-β for 0, 1, 2, and 3 days. (**A**) Western blot analysis of E-cadherin, N-cadherin, Vimentin, β-catenin, and Snail. β-actin served as a loading control; (**B**–**F**) the levels of the indicated protein was quantified with gray value (Mean ± SD, *n* = 3). * *p* < 0.05 compared with the corresponding group.

The process of EMT is always accompanied with down-regulation of epithelial cell markers and up-regulation of mesenchymal cell markers. As shown in Figure 2, the expression of epithelial cell marker, E-cadherin (E-cad), was significantly decreased with increasing stimulation of TGF-β. Meanwhile, the expressions of mesenchymal cell markers, N-cadherin (N-cad) and vimentin were positively associated with TGF-β in a time-dependent manner. β-catenin accumulates in complexes with cadherins at the cell membrane, which are involved in cell–cell interactions. Moreover, β-catenin translocates to the nucleus, interacts with transcriptional co-activators and induces the expression of EMT-associated genes. Due to the importance of the β-catenin, we verified the effects of TGF-β on the expression of β-catenin. Results showed that TGF-β elevated the expression of β-catenin. As a critical transcription factor, Snail initiated EMT through binding to the promoter region of E-cadherin directly or indirectly [34]. Here, in A549 cells, Snail was induced by TGF-β. In BEAS-2B cells, TGF-β also induced the EMT (Figure S1BF). The above results were in agreement with the EMT characteristics, suggesting a successful EMT model established in A549 cells.

### 3.3. 1α,25(OH)_2_D_3_ Opposes the Expression of EMT Markers and Extracellular Matrix Components Induced by TGF-β

**Figure 3:**
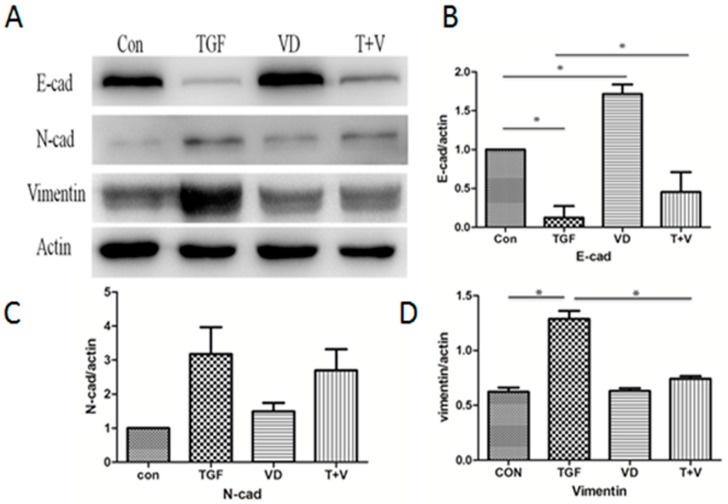

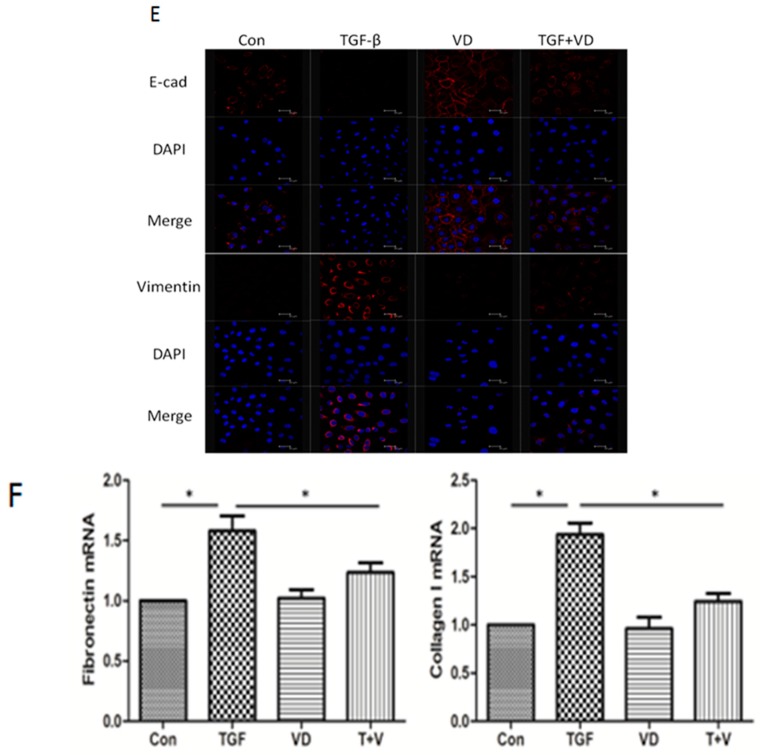
1α,25(OH)_2_D_3_ opposes the expression of EMT markers and extracellular matrix components induced by TGF-β. A549 cells were treated with 5 ng/mL TGF-β and/or 50 nmol/L 1α,25(OH)_2_D_3_ for 72 h. (**A**) Western blot analysis of E-cadherin, N-cadherin and Vimentin. β-actin served as a loading control; (**B**–**D**) The levels of the indicated protein was quantified with gray value (Mean ± SD, *n* = 3); (**E**) Representative pictures of indicated proteins E-cadherin, Vimentin were captured by confocal laser scanner microscopy. Nuclear DNA was visualized by DAPI staining (200× magnification); (**F**) qRT-PCR analyses of fibronectin and Collagen I (Mean ± SD, *n* = 3). * *p* < 0.05 compared with the corresponding group.

To investigate whether active vitamin D plays a role in inhibiting EMT, A549 cells were treated with 5 ng/mL TGF-β in the presence or absence of 1,25(OH)_2_D_3_, and EMT markers and extracellular matrix components were analyzed. Since 50 nM 1,25(OH)_2_D_3_ did not affect cell viability significantly (Figure S2), this concentration was selected for further investigation. As shown in Figure 3A–D, compared with TGF-β-treated cells, 1α,25(OH)_2_D_3_ increased the expression of E-cadherin, and decreased the expressions of N-cadherin and Vimentin. Immunofluorescence staining further demonstrated that 1α,25(OH)_2_D_3_ alleviated the TGF-β-induced EMT marker changes (Figure 3E). Furthermore, the changes were also observed in 1α,25(OH)_2_D_3_ treated alone cells. These findings indicated that 1α,25(OH)_2_D_3_ could reverse the TGF-β-induced EMT.

Collagen I and fibronectin, fundamental components of the extracellular matrix, are reported as biomarkers in pulmonary fibrosis. qRT-PCR suggested that 1α,25(OH)_2_D_3_ attenuated TGF-β-induced elevation of Collagen I and fibronectin (Figure 3F). Based on these results, we suggested that 1α,25(OH)_2_D_3_ attenuated TGF-β-induced pro-fibrotic effects through inhibition of EMT.

### 3.4. 1α,25(OH)_2_D_3_ Represses EMT-Related Transcription Factors by TGF-β and Increases VDR Expression

**Figure 4:**
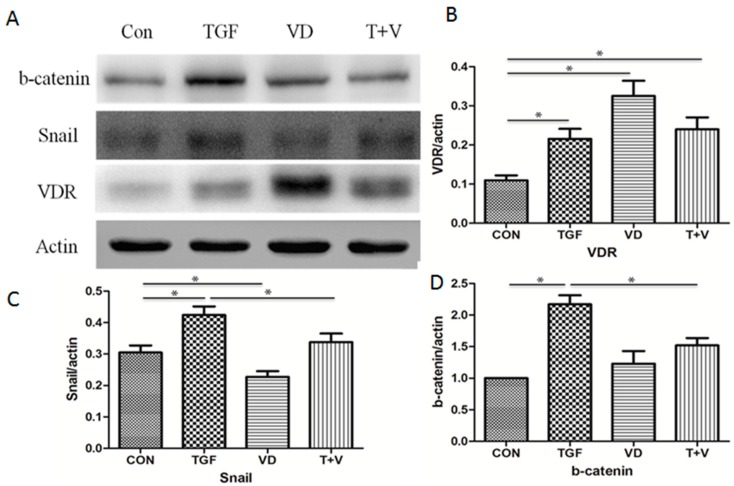
1α,25(OH)_2_D_3_ represses EMT-related transcription factors by TGF-β and increases VDR expression. A549 cells were treated with 5 ng/mL TGF-β and/or 50 nmol/L 1α,25(OH)_2_D_3_ for 72 h. (**A**) Western blot analysis of β-catenin, Snail and VDR. β-actin served as a loading control; (**B**–**D**) the levels of the indicated protein were quantified with gray value (Mean ± SD, *n* = 3). * *p* < 0.05 compared with the corresponding group.

In addition to the EMT markers, the EMT-related transcription factors were also assessed. Western blot analysis showed that 1α,25(OH)_2_D_3_ could decrease the induction of Snail and β-catenin by TGF-β. 1α,25(OH)_2_D_3_ mediated the target genes by binding to the vitamin D receptor. So, we investigated the expression of VDR using Western blot and found that 1α,25(OH)_2_D_3_ elevated the expression of VDR, compared with the TGF-β-treated cells (Figure 4). These results implied that VDR might be partly involved in attenuating TGF-β-induced EMT by 1α,25(OH)_2_D_3_.

### 3.5. 1α,25(OH)_2_D_3_ Prevents TGF-β-Induced Invasion and Metastasis in A549 Cells

**Figure 5:**
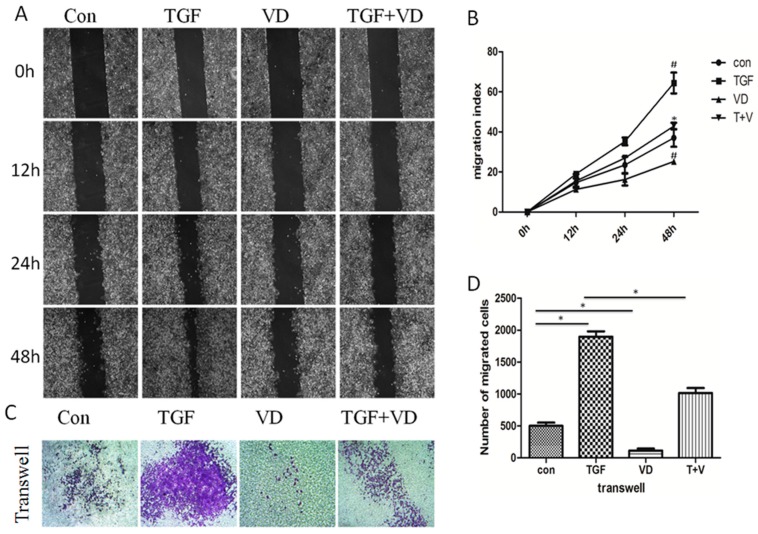
1α,25(OH)_2_D_3_ prevents TGF-β-induced invasion and metastasis in A549 cells. A549 cells were cultured in 0.5% FBS DMEM medium containing TGF-β and/or 1α,25(OH)_2_D_3_ for 48 h, then wound healing and transwell assay were performed. (**A**) Representative pictures of the wound area obtained at 0, 12, 24 and 48 h after scratching (100× magnification); (**B**) Migration index (%) = [(the initialized width of the scratch) − (the final width of the scratch)]/(the initialized width of the scratch). (**C**) Biocoat Matrigel Invasion Chambers (100× magnification); (**D**) Counts of cells under a microscope. ^#^ *p* < 0.05 compared with the control group, and * *p* < 0.05 compared with the TGF-β-treated cells.

Cells that underwent the EMT process usually displayed enhanced migration and invasion abilities, hence, wound healing and transwell assay were conducted in our study. As shown in Figure 5, after being treated with TGF-β, cells displayed higher migration and invasion abilities, whereas, 1α,25(OH)_2_D_3_ significantly repressed TGF-β-induced cell migration and invasion abilities.

## 4. Discussion

It is reported that hypovitaminosis D was common in patients with cystic fibrosis [35]. A few studies have reported the results of vitamin D supplementation for cystic fibrosis. However, the potential association between vitamin D and fibrosis has not been fully understood. In the present study, we have demonstrated that 1α,25(OH)_2_D_3_ abrogated the TGF-β-induced pro-fibrotic phenotype in A549 cells through the inhibition of EMT.

Increasing evidence has demonstrated that vitamin D and its analogue have suppression effects in fibrosis disease. In vivo, vitamin D inhibited UUO-induced kidney fibrosis and decreased thioacetamide-induced liver fibrosis [18,36]. Meanwhile, vitamin D inhibited proliferation and profibrotic maker expression in hepatic stellate cells and suppressed TGF-β-induced α-SMA and Collagen I expression in NRK-49F cells [37]. Preventive effects of 1α,25(OH)_2_D_3_ on pulmonary fibrosis were also observed in several models. Ramirez et al. demonstrated that vitamin D inhibited TGF-β-induced pro-fibrotic effects in lung fibroblasts and epithelial cells [33]. In addition, vitamin D treatment attenuated pulmonary fibrosis and associated cellular and ultra-structural changes induced by bleomycin in mice [38]. Due to the importance of inflammation and EMT in the development of pulmonary fibrosis, calcitriol can also inhibit bleomycin-induced early pulmonary inflammation and subsequent EMT in vivo [39]. Here, we suggested that vitamin D opposed the TGF-β-induced EMT and ECM-related genes in human alveolar epithelia cells.

In addition to its importance during embryonic development, the involvement of EMT in promoting organ fibrosis, tumor formation and cancer metastasis was becoming increasingly attractive. Classically, the changes of EMT markers in parallel with the morphological alteration were defined as features of EMT. TGF-β is known as an inducer of EMT and plays a critical role in pulmonary fibrosis. So, we establish an EMT model in TGF-β-induced A549 cells. Our experimental results suggested that TGF-β could alter A549 cells morphology from oval (epithelial morphology) to spindle (mesenchymal morphology). Meanwhile, the epithelial phenotype marker (E-cadherin) was repressed, while the mesenchymal phenotype marker (vimentin) was increased. However, 1α,25(OH)_2_D_3_ could reverse the expression levels of E-cadherin and vimentin induced by TGF-β, in parallel with the attenuation of cell migration and invasion capacities. In addition, 1α,25(OH)_2_D_3_ attenuated the TGF-β-induced alteration of cell morphology and EMT-related genes in BEAS-2S cells (Figure S3). In this way, we suggested that 1α,25(OH)_2_D_3_ could inhibit TGF-β-induced EMT, which may provide new clues in the clinical management of pulmonary fibrosis. Excessive deposition of extracellular matrix is a critical effector in pulmonary fibrosis. Collagen I and fibronectin are fundamental components of extracellular matrix. Therefore, we tested the effect of TGF-βon gene expressions in A549 cells. We observed that 1α,25(OH)_2_D_3_ significantly impaired TGF-β-induced increases of Collagen I and fibronectin, which suggested the anti-fibrotic effects of 1α,25(OH)_2_D_3_.

The changes in gene expression that contribute to the repression of the epithelial phenotype and activation of the mesenchymal phenotype involve regulators, including Snail. Snail is a kind of zinc-finger protein, and represses epithelial genes by binding to E-box DNA sequences [40]. In addition to repressing epithelial genes, Snail activates genes that contribute to the mesenchymal phenotype. The increased expression of Snail was reported in a previous study of patients with fibrosis disease [41]. Our results showed that 1α,25(OH)_2_D_3_ attenuated the TGF-β-induced up-regulation of Snail in alveolar epithelial cells, which might be involved in the inhibition of TGF-β induced EMT. Multiple signaling pathways manipulate the EMT process, including the Wnt/β-catenin signaling pathway. Activated β-catenin is accumulated in the cytoplasm, and resulted in the activation of target genes. Studies have provided evidence that TGF-β promoted the β-catenin nuclear translocation while 1α,25(OH)_2_D_3_ repressed the Wnt/β-catenin signaling pathway. Our present study found that TGF-β stimulated the total expression of β-catenin in a time-dependent manner and the combination of 1α,25(OH)_2_D_3_ and TGF-β constrained the expression the β-catenin.

The active metabolite of vitamin D, 1α,25(OH)_2_D_3_, exerts biological and physiological functions via VDR, which is a ligand-dependent transcription factor. VDR has been described in the fetal lung, as the immature lung is known as a target organ for 1α,25(OH)_2_D_3_. Moreover, VDR was recently detected in the epithelia of normal and malignant human bronchial tissue, while vitamin D metabolites stimulated the expression of VDR responsive genes in lung epithelial cells [42]. Consistent with Ramirez’s study, we observed the up-regulation of VDR in A549 cells treated with 1α,25(OH)_2_D_3_. In Ramirez’s study, 1 µM 1α,25(OH)_2_D_3_ was chosen as the highest dose based on several in vitro studies in fibroblasts and cancer cells. However, in our study, we found that 1 µM 1α,25(OH)_2_D_3_ caused significant cytotoxicity, and 50 nM was selected for the investigation. In addition, we previously found that 1,25(OH)_2_D_3_ could inhibit cell growth at a dose of 50 nM or higher in other cell lines [43]. We suggested that the origin and species of cells might account for the differences observed between the two studies. In this way, considering the possible cytotoxicity of a high dose of vitamin D, we proposed that the appropriate concentration for vitamin D application should vary based on the purpose of the study.

## 5. Conclusions

In conclusion, our study demonstrated that 1α,25(OH)_2_D_3_ could suppress the EMT program induced by TGF-β, which may account for its inhibitory effects on the invasion and migration of human alveolar epithelia cells. These findings revealed a potential novel target for fibrotic lung disorders.

## Figures and Tables

**Table 1:** Primer sequences for reverse transcription-quantitative polymerase chain reaction.

Gene Name	Forward Sequence (5′-3′)	Reverse Sequence (5′-3′)
Collagen I	ACGTCCTGGTGAAGTTGGTC	ACCAGGGAAGCCTCTCTCTC
Fibronectin	GAGCTATTCCCTGCACCTGA	CGTGCAAGGCAACCACACT
GAPDH	CGTGCAAGGCAACCACACT	TGGCAGGTTTTTCTAGACGG

## Supplementary Materials

The following are available online at www.mdpi.com/2072-6643/9/9/980/s1.
