# Supplementary material for: 1α,25-dihydroxyvitamin D3 Attenuates TGF-β-Induced Pro-Fibrotic Effects in Human Lung Epithelial Cells through Inhibition of Epithelial–Mesenchymal Transition
Source: Nutrients. 2017 Sep 6;9(9):980. doi: 10.3390/nu9090980 (PMC5622740; doi:10.3390/nu9090980)
Supplement: Supplementary file 1 [file nutrients-09-00980-s001.pdf]

Supplementary Data

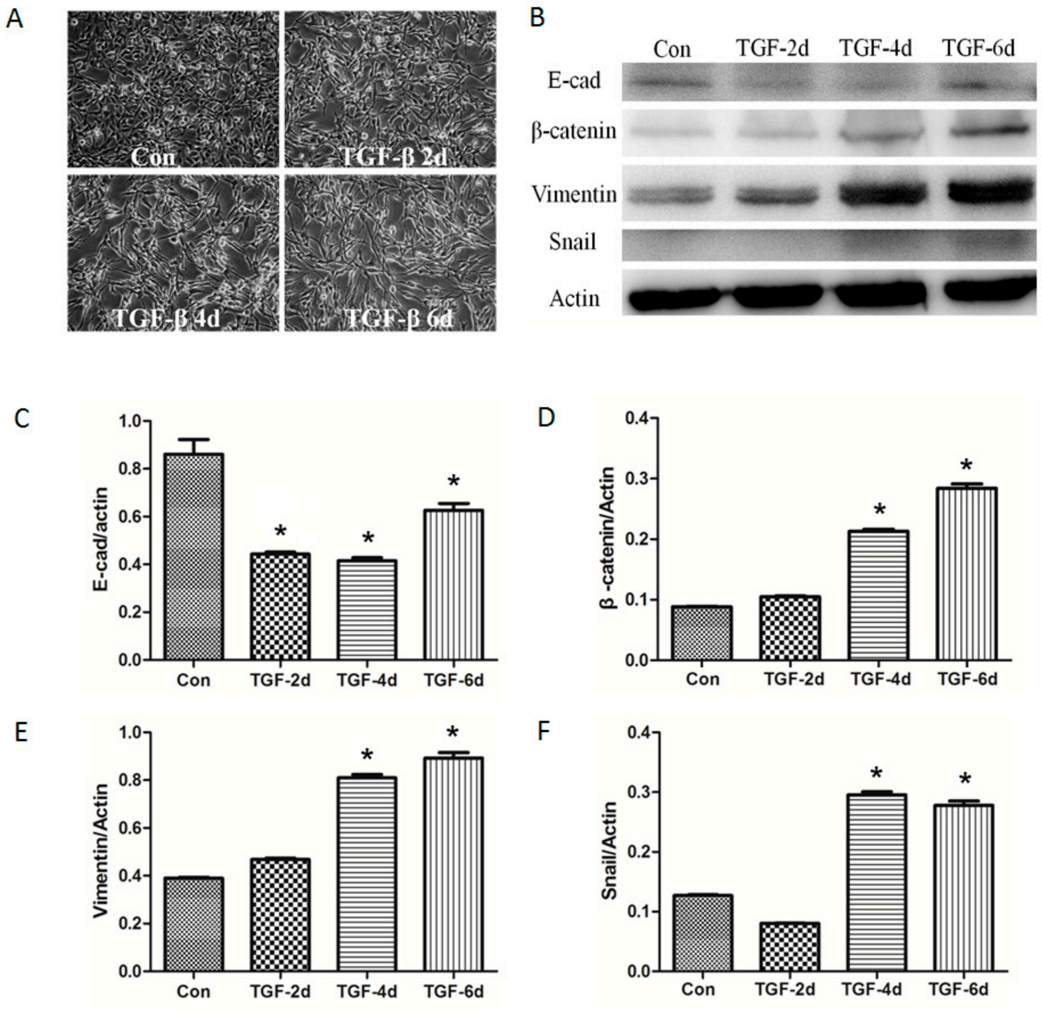

Figure S1. TGF-β induced EMT in BEAS-2B cells.

BEAS-2B cells were exposed to 5 ng/ml TGF-β for 0, 2, 4, and 6 days. (A) Morphological features of cells at the last day (400×magnification); (B) Western blot analysis of E-cadherin, Vimentin, β-catenin, and Snail. β-actin served as a loading control; (C-F) the levels of the indicated protein was quantified with gray value (Mean±SD, n=3). $^{*}P < 0.05$ compared with the corresponding group.

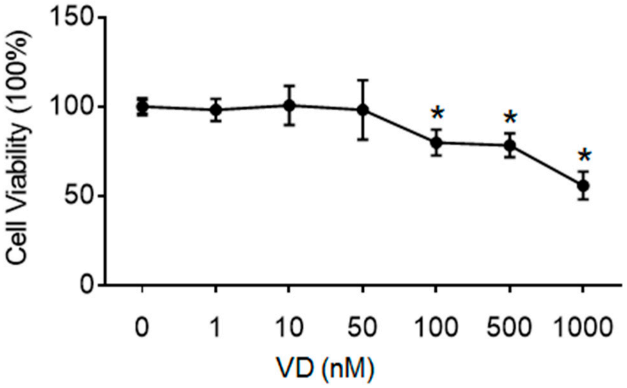

Figure S2. Effects of 1α,25(OH)$_2$D$_3$ on cell viability in A549 cells.

A549 cells were exposed to different concentration of 1α,25(OH)$_2$D$_3$ (0, 1, 10, 50, 100, 500, 1000 nmol/L) for 3 days. Then 10 μl of CCK-8 solution was added and the cells were incubated for 1 h. Absorbance was measured at 450 nm in a microplate reader (BioTec Instruments, Inc., Winooski, VT, USA) and cell viability was calculated. Each experiment was performed in triplicate. $^{*}P < 0.05$ compared with the corresponding group.

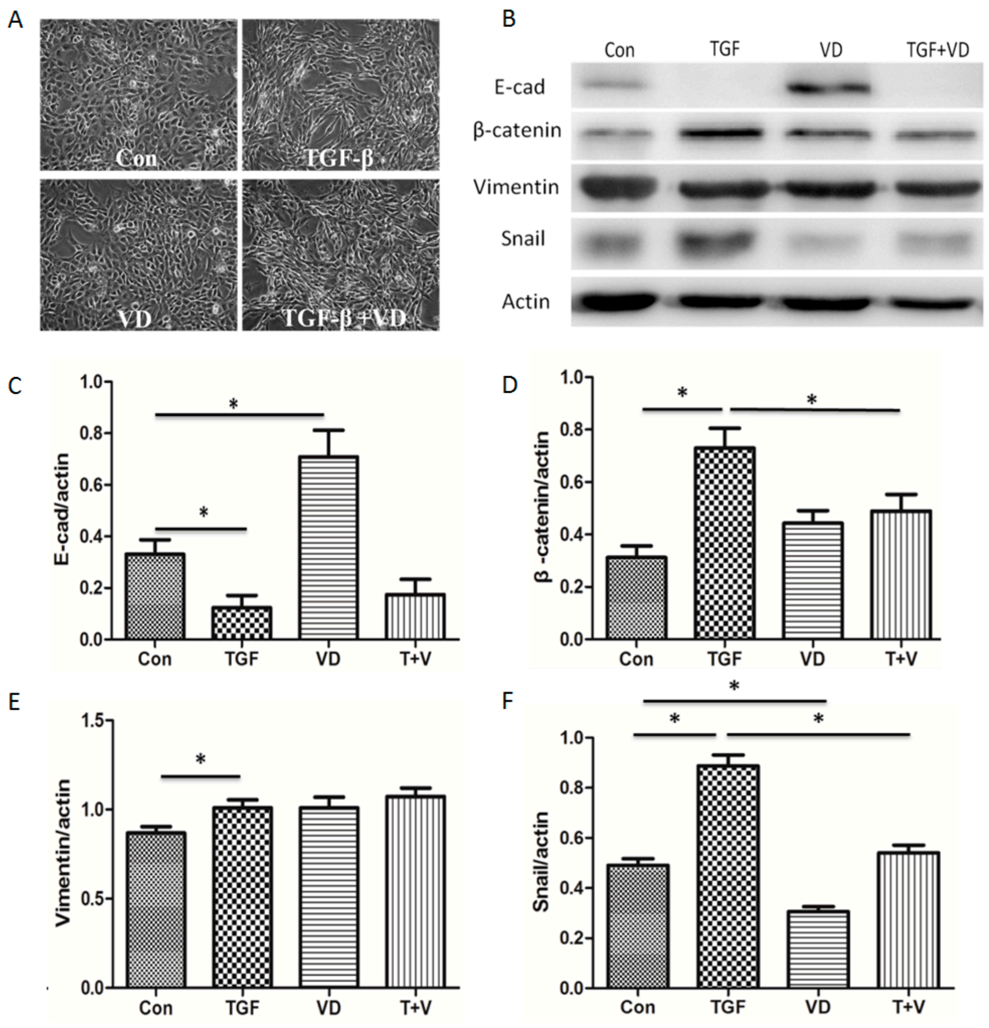

Figure S3. Vitamin D prevented TGF-β induced EMT markers alteration in BEAS-2B cells.

BEAS-2B cells were treated with 5 ng/ml TGF-β and/or 50 nmol/L 1α,25$(OH)_2D_3$ for 6 days. (A) Morphological features of cells at the last day (400×magnification); (B) Western blot analysis of E-cadherin, Vimentin, β-catenin, and Snail. β-actin served as a loading control; (C-F) The levels of the indicated protein was quantified with gray value (Mean±SD, n=3). $^{*}P < 0.05$ compared with the corresponding group.
